# Effect of Lactose Pseudopolymorphic Transition on the Aerosolization Performance of Drug/Carrier Mixtures

**DOI:** 10.3390/pharmaceutics11110576

**Published:** 2019-11-04

**Authors:** Andrea Della Bella, Michele Müller, Andrea Danani, Luciano Soldati, Ruggero Bettini

**Affiliations:** 1 Food and Drug Department, University of Parma, 43124 Parma, Italy; dellabella.andre@gmail.com; 2 Micro-Sphere S.A., Ponte Cremenaga, 6996 Monteggio, Switzerland; mmul@sferalp.com (M.M.); luciano.soldati@bluewin.ch (L.S.); 3 Istituto Delle Molle di Studi Sull’Intelligenza Artificiale, Scuola Universitaria Professionale Della Svizzera Italiana, 6928 Manno, Switzerland; andrea.danani@idsia.ch

**Keywords:** lactose, DPI, polymorphism, aerosolization performance

## Abstract

Physico-chemical properties of lactose are key factors in adhesive mixtures used as dry powder inhaler (DPI). Despite the abundant literature on this topic, the effect of the polymorphism and pseudo-polymorphism of lactose has been seldom investigated and discussed although often lactose used in DPI is subjected to unit operations, which may alter its solid-state properties. Here, we studied the aerosolization performance of salbutamol sulphate (SS) or budesonide (BUD) formulations by investigating the effect of lactose pseudopolymorphism in ternary (coarse lactose/fine lactose/drug) and binary (coarse lactose/drug) mixtures. An improvement of the aerosolization performance of SS formulations with the increase of the amount of fine micronized lactose up to 30% (fine particle fraction (FPF) = 57%) was observed. Micronized lactose contained hygroscopic anhydrous α-lactose, which converted to α-lactose monohydrate at ambient conditions. This implied that the positive effect of fines on the aerosolization performance decreased and eventually disappeared with the formulation aging. Positive effect on SS deposition was observed also with binary mixtures with anhydrous lactose, whereas the opposite occurred with budesonide-containing formulations. The collected data demonstrated the crucial role of the carrier crystal form on the positive effect of fines on the deposition.

## 1. Introduction

Dry powder inhalers (DPIs) are routinely used for the treatment of respiratory diseases and are composed of a powder formulation in an inhaler device [1,2]. In order to achieve deep lung penetration, active pharmaceutical ingredients (APIs) are usually micronized to sizes between 1 and 5 μm resulting in highly cohesive particles with poor flow properties [3]. Therefore, to improve handling and dispersibility, drug particles are generally blended with coarser carrier particles to which they adhere forming “adhesive” mixtures [4]. The interparticulate forces occurring within such systems have to be sufficiently strong to afford a stable and homogeneous mixture, but at the same time weak enough to allow the separation of the drug and the carrier upon aerosolization. The detachment of drug particles from the carrier is crucial in determining DPI performance and strongly depends on the surface characteristics of both components. Thanks to the fact that it is non-toxic, inexpensive, and compatible with the majority of low molecular weight drugs, lactose (in particular α-lactose monohydrate (Lα·H_2_O)) is the most commonly used carrier in dry powder formulations for inhalation [3]. Since lactose represents, by far, the most abundant component in the formulation, the effect of its properties on DPI performance has been the core theme of a large number of studies [5]. Several parameters, such as the carrier particle size [6,7,8], morphology [9,10], roughness [9,11,12,13,14], physico-chemical properties [15,16], polymorphism [17], and the presence of fines [18,19,20,21] have been studied in order to understand their influence on the DPIs performance. The heterogeneity and complexity of these variables, which are often related to each other, make the understanding of dry powders behaviour particularly complicated and still an open issue [3,4,5,22,23].

The addition of extra lactose fines proved to be a suitable strategy to improve the aerosolization performances [21]. This aspect has been rationalized by developing two main theories: the active sites hypothesis and the agglomeration hypothesis. In addition to these “traditional” explanations, other possible mechanisms have been recently proposed [24].

Inhalation grade lactose is often subject to unit processes, such as spray drying, milling, or micronization, which may alter its physico-chemical and solid-state properties [3,23]. For example, spray drying is likely to produce changes in the anomeric composition of lactose as a consequence of the mutarotation occurring in the feed solution before/during the process [25]. Milling and micronization may result in the formation of amorphous material [26,27,28]. The use of milled lactose instead of untreated or sieved lactose improved DPIs performance, but the improvement was generally attributed to the presence of a higher amounts of intrinsic fines in the sample of milled lactose used as carrier [18].

In a previous work, we have put into evidence the effect of the micronization process on the solid-state properties of the lactose fines underlying the pseudo-polymorphic transition of the micronized lactose with partial formation of hygroscopic anhydrous α-lactose rather than amorphous lactose [29].

Few works specifically focused on the effect of polymorphic or pseudopolymorphic form influence on API aerosolization performance [17,30]. However, in both cases, the effect of the lactose solid state could not clearly be separated from that of a different percentage of intrinsic fines in the tested lactose samples. 

In the present work, the aerosolization performance of lactose/salbutamol sulphate and lactose budesonide formulations was studied by focusing on the solid-state properties of lactose. In particular, we investigated the effect of the pseudopolymorphic transition of lactose stemming either from the micronization or thermal dehydration process both in ternary (coarse lactose/fine lactose/API) and binary (coarse lactose/API) mixtures, aiming at investigating whether the well-documented positive effect of fines on the respirability performance could be ascribed solely to the dimension of fine lactose particles [31] or also to a specific characteristic of the solid-state of the lactose fine particle surface which is a direct consequence of the micronization process. 

Micronized salbutamol sulphate, SS (logP = −1.3) [32] and budesonide, BUD (logP = 3.20) [33] were taken as model active pharmaceutical ingredient (API) with hydrophilic and lipophilic characteristics, respectively.

## 2. Materials and Methods

### 2.1. Materials

Coarse Lα·H_2_O (dv_50_ = 91.7 µm) was supplied by Kerry (Tralee, Ireland). Lacto-Sphere^®^ MM50 (sieved Lα·H_2_O, dv_50_ = 53.1 µm) was provided by Micro-Sphere SA (Ponte Cremenaga, Switzerland). Micronized salbutamol sulphate (d_V50_ = 2.7 µm) was supplied by Teva Pharmaceuticals Industries (Petach Tikva, Israel) and budesonide (d_V50_ = 1.9 µm) supplied by Plantex Chemicals B.V. (Utrecht, Netherlands).

### 2.2. Methods

#### 2.2.1. Production of Micronized Lactose 

Micronized lactose (d_V50_ = 2.1 µm) was produced by micronizing coarse Lα·H_2_O with a J-70 fluid jet micronizer (Tecnologia Meccanica, Albegno di Treviolo, Italy) fed with nitrogen. The process parameters were set as follows: Venturi pressure P_V_ = 10 bar, ring pressure P_R_ = 10 bar, feed rate R_F_ = 1 kg h^−1^.

#### 2.2.2. Preparation of Hygroscopic Anhydrous α-Lactose (Lα_H_)

Lα_H_ was prepared following a procedure reported elsewhere [29] by heating Lacto-Sphere^®^ MM50 at 120 °C for 9 h under a dry nitrogen flow. After production, Lα_H_ was always handled in a glove-box filled with dry nitrogen (RH < 5%) in order to prevent possible rehydration.

#### 2.2.3. Preparation of Stable Anhydrous α-lactose (Lα_S_)

Lα_S_ was prepared as described in a previous work [29] by heating Lacto-Sphere^®^ MM50 at 145 °C for 6 h under a dry nitrogen flow.

#### 2.2.4. Differential Scanning Calorimetry (DSC)

DSC measurements were performed using an Indium calibrated DSC 821e instrument (Mettler Toledo, Schweiz, Switzerland) driven by STARe 11/2014 (Mettler Toledo). DSC traces were recorded by placing accurately weighed quantities (6–12 mg) of powder sample in a 40 μL Aluminium pan, which was then sealed and pierced twice. Scans were performed between 25 °C and 250 °C at a scanning rate of 10 °C min^−1^ under a purging nitrogen atmosphere (100 mL min^−1^).

#### 2.2.5. Particle size Determination

The dimensional distribution of lactose samples and APIs was determined by laser light diffraction using a Spraytec analyzer (Malvern, UK). Two different procedures were adopted.

In the first case, a supersaturated solution of lactose in ethanol was prepared by adding 5 g of lactose to 1 L of ethanol (96% *v*/*v*). The solution was stirred overnight and then filtered under vacuum through a nylon membrane filter (0.45 µm, Ø 47 mm, Whatman^®^, GE Healthcare Life Sciences, Little Chanfont, UK). The filtered solution was used as dispersing liquid for the analysis. Furthermore, 100 mg of each sample were dispersed in 10 mL of the lactose-saturated ethanol solution and put in an ultrasound bath (40kHz) for 5 min before the analysis. Each measurement was performed in triplicate with an 8% obscuration threshold. To analyze the APIs, cyclohexane (VWR, Ismaning, Germany) was chosen as dispersing liquid. Then, 10 mg of drug were dispersed in a 0.1% *w*/*v* solution of Span^®^ 85 Croda Inc., Newark, NJ, USA) in cyclohexane and sonicated for 5 min before being analyzed. Each measurement was repeated three times with an 8% obscuration threshold.

#### 2.2.6. Scanning Electron Microscopy (SEM)

The morphology and surface roughness of lactose carriers were evaluated by scanning electron microscopy using a FESEM SUPRA™ 40 (Carl Zeiss, Germany). Each powder sample was deposited on adhesive black carbon tabs pre-mounted on aluminum stubs so as to allow the dispersion of the charge and exposed to a gold metallization process to deposit a gold film of 60 nm. The particles in excess were removed by a gentle nitrogen flow. The samples were analyzed under high vacuum conditions (1.33 × 10^−2^ Pa for 30 min) and the images were collected at different magnifications using an accelerating voltage of 1.5 or 2 kV.

#### 2.2.7. Preparation of Drug/Carrier Mixtures

Mixtures containing 1% *w*/*w* API were blended with a Turbula^®^ T2A shaker-mixer (WAB, Switzerland) at 30 rpm using a stainless-steel vessel (internal volume = 240 mL, internal diameter = 5 cm, height = 12 cm), which was grounded to prevent the accumulation of electrostatic charges. Six grams of each binary mixture were prepared according to a three steps procedure: (i) Mixing of 0.6 g of carrier with the API for 30 min; (ii) addition of 1.8 g of carrier followed by a 30 min mixing step; and (iii) addition of the remaining portion of carrier and mixing for 1 h. In the case of ternary mixtures, the above-described procedure was preceded by a first step during which coarse and fine lactose were blended for 30 min.

#### 2.2.8. Salbutamol Sulphate Quantification

The quantitative determination of salbutamol sulphate was performed by UV spectroscopy. Measurements were carried out with a V-570 UV-Vis/NIR spectrophotometer (Jasco, Japan). Each powder sample was dissolved in water and the absorbance was determined at λ = 224 nm using quartz cuvettes with optical path length of 1 cm. For each sample, three quantitative determinations were performed, each of which was obtained as the average of 10 consecutive readings. The analytical method was validated as regards to linearity of the response (absorbance vs. concentration) in the concentration range 5–64 μg mL^−1^, limit of detection (0.57 µg mL^−1^), and limit of quantification (1.91 µg mL^−1^) evaluated as 3.3 x σ/slope and 10 x σ/slope, respectively, where σ and slope are the standard deviation of the intercept and the slope, respectively, of the of the regression line of the absorbance vs. concentration experimental points. An additional calibration curve (absorbance vs. concentration) was built to evaluate the UV response of the capsules used in the in vitro deposition tests. The linearity of the response was assessed in the concentration range 2–14 mg mL^−1^ (LOD = 0.57 mg mL^−1^, LOQ = 1.89 mg mL^−1^) using water as solvent.

#### 2.2.9. Budesonide Quantification

The quantitative analysis of budesonide was performed by HPLC. A system composed of two LC-10AT VP pumps (Shimadzu, Tokyo, Japan), an SPD-M10A VP diode array detector (Shimadzu), a CTO-10AS VP oven column (Shimadzu), and a Waters 717 plus autosampler (Waters, Milford, MA, USA) was used. The analysis was carried out at 25 °C using a Nova-Pak C-18 column (4 µm, 3.9 mm x 150 mm, Waters, Milford, MA, USA). Budesonide-containing solutions were isocratically eluted at a flow of 0.6 mL min^−1^ employing a 6:4 *v*/*v* acetonitrile/water solution as mobile phase. An injection volume of 50 µL, a run time of 6 min, and a wavelength of 254 nm were set for the analysis. The analytical method was validated in terms of linearity of the response (peak area vs. concentration) in the concentration range 0.3–26.6 µg mL^−1^ (LOD = 0.026 µg mL^−1^, LOQ = 0.087 µg mL^−1^) using a 6:4 *v*/*v* acetonitrile/water solution as solvent.

#### 2.2.10. Homogeneity Test

The homogeneity of drug/carrier mixtures was checked at the end of the mixing procedure. For each mixture five samples (20 mg each) were collected from five different spots of the powder bed. Each sample was dissolved in 10 mL of an appropriate solvent (water in the case of salbutamol sulphate, and a 6:4 *v*/*v* acetonitrile/water solution in the case of budesonide) and the quantification of the API was performed. Homogeneity was assumed when the coefficient of variation (calculated as the percentage of the ratio of the standard deviation to the mean value on the five measurements) was below 5%.

#### 2.2.11. In Vitro Deposition Tests

In Vitro aerodynamic assessment was performed using a next generation impactor (NGI, Copley Scientific, Nottingham, UK) equipped with a pre-separator. In order to control particle rebound, the first seven stages of the impactor were coated with a suitable filming solution and then allowed to dry prior to use. A 1% *w*/*w* solution of glycerol in methanol was employed when mixtures containing salbutamol sulphate were tested, whereas a 1% *w*/*v* solution of Span^®^ 85 in cyclohexane was used for mixtures containing budesonide. The terminal stage (micro-orifice collector, MOC) of the impactor was fitted with a glass fiber filter (Type A/E, Pall Corporation, Port Washington, NY, USA). The central cup of the pre-separator was filled with 15 mL of water in the case of salbutamol sulphate, or a 6:4 *v*/*v* acetonitrile/water solution in the case of budesonide. After completing the assembly, the NGI was connected to a VP 1000 vacuum pump (Erweka, Langen, Germany) and the flow rate through the impactor was checked using a mass flowmeter (model 3063, TSI, Shoreview, MN, USA). A low resistance single-dose DPI, Turbospin^®^ #2 (PH&T, Milan, Italy), was chosen as aerosolization device. For each mixture, 10 Quali-V^®^ capsules size 2 (Qualicaps^®^ Europe, Madrid, Spain) were filled with 20.0 ± 0.1 mg of powder, introduced in the inhaler device, and finally, pierced. Once the device was connected to the impactor through an airtight rubber mouth, the vacuum pump was activated at a flow of 70 L min^−1^ for 3.4 s so that 4 L of air were drawn through the apparatus (Ph. Eur. 9, 2.9.18). For each mixture, 10 consecutive aerosolizations were performed. At the end of the deposition experiment, the NGI was disassembled and two different procedures were adopted depending on the API under examination.

Salbutamol sulphate deposited on each stage of the impactor was recovered with aliquots of water, which were finally transferred into volumetric flasks of adequate volume. The obtained solutions were filtered through a cellulose acetate syringe filter (porosity 0.45 µm, GVS Filter Technology, Findlay, OH, USA) before being analyzed. A volumetric flask named *device* was used to collect the salbutamol sulphate remained in the Turbospin^®^ device and the capsules, which were dissolved in water at the end of the experiment in order to ensure complete recovery of the drug. Therefore, in this case, the absorbance value initially recorded was corrected by subtracting the contribution due to the absorbance of the Quali-V^®^ capsules.

Budesonide was recovered from each stage of the impactor with appropriate volumes of a 6:4 *v*/*v* acetonitrile/water solution. Before being transferred into vials for the HPLC analysis, the solutions were filtered through a regenerated cellulose syringe filter (porosity 0.45 µm, Analytical Technology, Italy).

All the mixtures were tested in triplicate immediately after preparation. Their aerodynamic performance was evaluated by calculating: The emitted dose (ED), obtained as the sum of the portions of drug recovered from the mouthpiece adapter, the induction port, the pre-separator, and all the stages of the impactor and expressed in micrograms; the fine particle dose (FPD), namely the quantity of drug with a cut-off diameter lower than 5 µm, calculated by interpolation according to the European Pharmacopoeia (Ph. Eur. 9, 2.9.18) and expressed in μg; the fine particle fraction (FPF), calculated as the ratio of the FPD to the ED and expressed as percentage.

#### 2.2.12. Statistical Analysis

The statistical analysis was performed with Microsoft Office Excel 16.16.13 (Microsoft Corp., Redmond, WA, USA) employing a two-tailed unpaired t-test with significance level fixed at *p*-value = 0.05. Experimental variability has been always expressed as standard deviation.

## 3. Results and Discussion

### 3.1. Characterization of Lactose Samples

The solid-state of lactose carriers (Lα·H_2_O, Lα_H_, and Lα_S_) and fine micronized lactose was previously investigated by XRPD and DSC [29]. Micronized lactose was characterized by a lower degree of crystallinity compared to the coarse Lα·H_2_O used as starting material for the micronization process. This effect was attributed to the presence of a certain amount of Lα_H_ and amorphous lactose, which were generated as a consequence of the mechanical stress stemming from the micronization. In the same work, the anomeric composition of each lactose sample was also determined by ^1^H NMR spectroscopy according to the method proposed by Jawad et al. [25]. The thermal dehydration process used to prepare the anhydrous forms of lactose led to the formation of limited amounts of β-lactose. In all the other cases, the percentage of β-form was negligible and revealed that micronization in the reported conditions did not induce mutarotation [29].

The same lactose carriers were used in the present work and further characterized in terms of particle size distribution (Table 1).

The carriers exhibited similar and not statistically different d_V50_ and d_V90_ (*p* > 0.05 in all cases), but different d_V10_, with the anhydrous forms containing lower amounts of intrinsic fines, which probably coalesced during thermal dehydration to form slightly larger particles.

**Figure 1:**
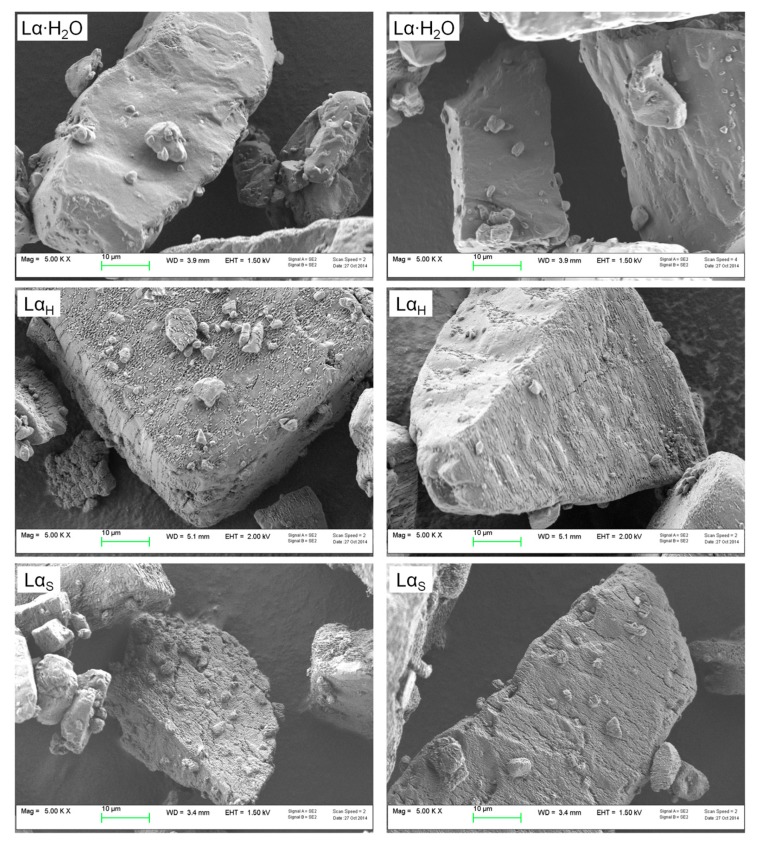
SEM micrographs of lactose carriers at 5000× magnification: α-lactose monohydrate (as received), Lα·H_2_O; hygroscopic anhydrous α-lactose, Lα_H_; stable anhydrous α-lactose, Lα_S_.

Visual examination of lactose carriers (Figure 1) showed particles with similar size and shape, but different surface characteristics.

The surface of Lα·H_2_O particles was irregular, but smoother than that of the other polymorphs. Roughness increased in Lα_H_ and Lα_S_ particles, which showed more clefts and defects, likely ascribable to the higher temperature used for the preparation of the stable polymorph.

Overall, the two treated powders appeared quite similar as to surface morphology although the general tomahawk shape was not changed compared to lactose as received. Both treated samples presented a slightly corrugated surface likely stemming from the partial evaporation of water. 

### 3.2. Effect of Lactose Solid-State on Drug Respirability

#### 3.2.1. Ternary Mixtures

Ternary mixtures containing 1% *w*/*w* salbutamol sulphate (SS), Lα·H_2_O as carrier and variable percentages of micronized lactose were prepared and compared to a binary mixture containing no fines in order to evaluate the effect of the presence of lactose fines on the respirability of the drug (Table 2).

As the percentage of lactose fines in the mixtures increased, a progressively reduced homogeneity was observed. In the case of mixture M-50, an additional mixing step of 120 min was necessary to achieve the fixed level of homogeneity (CV < 5%).

The aerosolization performance of each mixture was investigated in terms of emitted dose (ED), fine particle dose (FPD, the dose of particle with aerodynamic diameter lower than 5 µm), and fine particle fraction (FPF) (Table 3).

The presence of fine lactose had a remarkably positive effect on the deposition of salbutamol sulphate. In agreement with literature data [21], the ternary mixtures afforded FPDs and FPFs higher than that obtained with the binary mixture containing no added fines (M-SS). In particular, the best results were achieved with mixture M-30, which gave an FPD almost four times greater than that provided by mixture M-SS. On the other hand, the addition of the highest amount of fines (mixture M-50) resulted in a lowering of the ED due to the formation of agglomerates, which remained in the capsules during inhalation as well as in a lower FPD compared to the mixtures M-10 and M-30.

**Figure 2:**
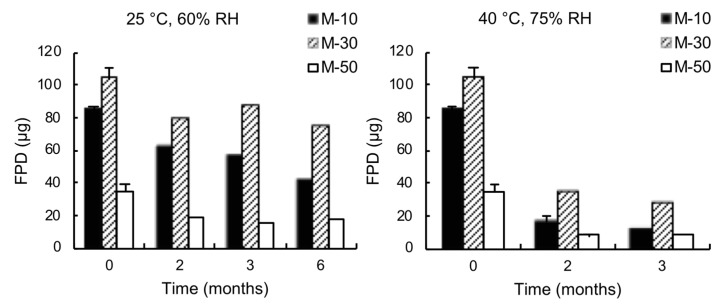
FPD of salbutamol sulphate from ternary mixtures containing 10% *w*/*w* (M-10), 30% *w*/*w* (M-30), and 50% *w*/*w* (M-50) of micronized lactose stored at 25 °C, 60% RH (left) and 40 °C, 75% RH (right). The bars represent the standard deviation (*n* = 5).

The stability of the ternary mixtures was evaluated by storing them in glass vials stoppered with high-density PET caps under two different conditions of temperature and relative humidity: 25 °C, 60% RH and 40 °C, 75% RH. Deposition tests were repeated after two, three, and six months for the first storage condition and after two and three months for the second one. Figure 2 shows the FPD obtained from the aged mixtures.

**Figure 3:**
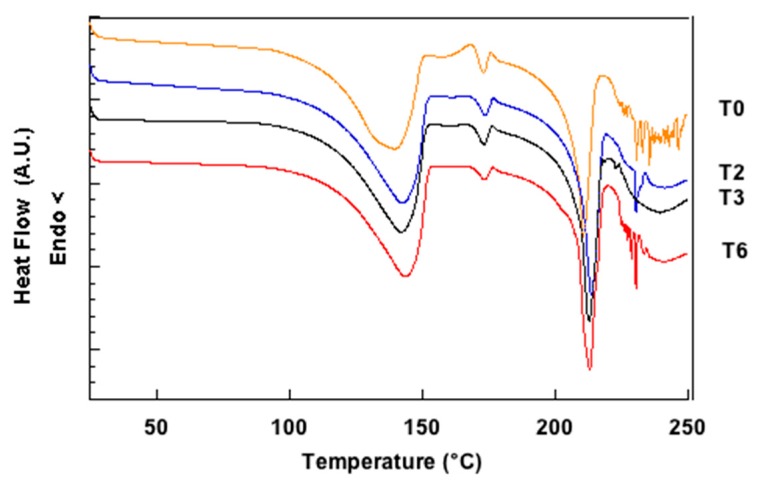
Differential scanning calorimetry (DSC) traces of micronized lactose recorded immediately after production (T0), after two (T2), three (T3), and six (T6) months storage at 25 °C, 60% RH.

Aging led to a progressive worsening of the aerosolization performance, especially when the mixtures were stored at 40 °C, 75% RH. The storing conditions were such that the powder could be influenced not only by the temperature, but also by the relative humidity as the vials were stoppered but not tightly sealed with the PET caps. The absorption of moisture may have led to increased capillary forces between the adhering particles within the mixtures, thus resulting in a more difficult detachment of the drug during aerosolization [34]. Moreover, unfavorable interactions between lactose and drug might have developed due to the fact that the surface of lactose fines was partly composed of hygroscopic anhydrous α-lactose and amorphous lactose, which are unstable and continuously evolved over time. In this respect, Figure 3 shows the evolution of the DSC trace of micronized lactose during aging. DSC measurements were performed alongside the in vitro deposition tests on samples of micronized lactose stored at 25 °C, 60% RH.

The thermal events in the region between the evaporation of crystalline water (peak at 143 °C) and the melting of lactose (peak at 218 °C) progressively decreased in intensity during aging. This was indicative of the conversion of hygroscopic anhydrous α-lactose and amorphous lactose mainly into α-lactose monohydrate (although the possible formation of small amounts of mixed α/β compounds could not be excluded a priori) [29]. Such transformations would be obviously favored under higher RH conditions. These results strongly suggested that a correlation between the nature of the solid-state of micronized lactose and the aerosolization performance is likely to exist. Based on these findings we hypothesized that, differently from what is commonly reported in the literature [24], the positive effect of lactose fines would depend not only on their reduced size, but also on a specific characteristic of their solid-state leading to the decrease of the surface interaction with the drug. To check the validity of such hypothesis, different lactose polymorphs were selected to be employed as carriers in the preparation of binary mixtures.

#### 3.2.2. Binary Mixtures

**Figure 4:**
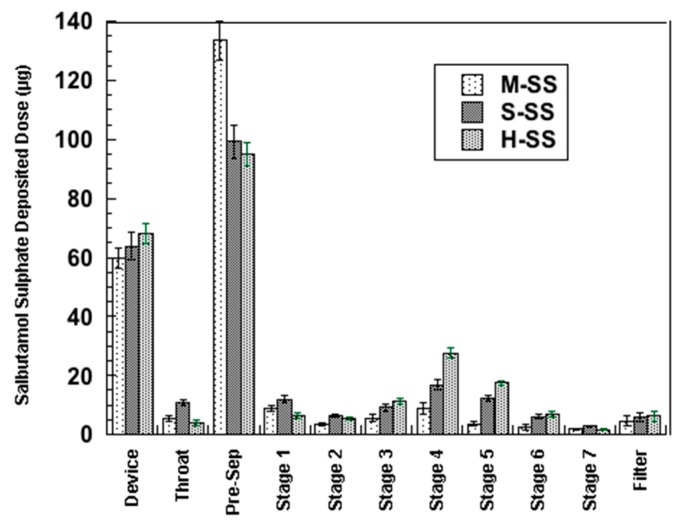
Salbutamol sulphate deposition profile from binary mixture with α-lactose monohydrate (M-SS), stable anhydrous α-lactose (S-SS), and hygroscopic anhydrous α-lactose (H-SS) with an next generation impactor (NGI) apparatus. The bars represent the standard deviation (*n* = 5).

Reasonably being the major component on the surface of micronized lactose particles, Lα_H_ was selected as one of the alternative carriers to be tested. Lα_S_ was chosen because of its anhydrous character associated with a higher stability. Therefore, Lα_H_ and Lα_S_ were used to prepare two binary mixtures, H-SS and S-SS, containing 1% w/w salbutamol sulphate. The results of the in vitro deposition tests are reported in Figure 4 in comparison with those obtained from the binary mixture containing Lα·H_2_O as carrier (M-SS). 

Table 4 summarizes the aerosolization parameters obtained from the two binary mixtures containing either hygroscopic or stable anhydrous lactose.

Considering the data obtained with the binary mixture containing Lα·H_2_O as carrier (mixture M-SS, see Table 3) the use, as carriers, of the anhydrous forms of α-lactose afforded a remarkable positive effect on the deposition of salbutamol sulphate. These data are in agreement with those reported by Larhrib et al. [15] who studied the effect of different lactose grade with particle size distribution 63–90 µm on the aerosolization performance of salbutamol sulphate and evidenced that formulation containing anhydrous β-lactose afforded a FPF approximatively two times higher than formulation containing regular lactose. Opposite results were, instead, reported by Traini et al. [17]. However, these authors reported SS aerosolization performance for α-lactose monohydrate compared to the α- anhydrous form with the latter containing significantly lower amounts of intrinsic fines. Furthermore, the crucial role of the device in determining the detachment of the API from the carrier surface cannot be disregarded. 

These findings reported here underline the key role of lactose solid-state in affecting the aerosolization performance. In both cases (H-SS and S-SS), the obtained FPF was significantly higher than that provided by Lα·H_2_O. In particular, the mixture with Lα_H_ gave ED, FPD, and FPF close to those achieved with the ternary mixture containing 10% of fines (mixture M-10). It is worth underscoring that the anhydrous α-lactose polymorphs provided better results with respect to the hydrate form, despite the fact that they contained a significantly lower amount of intrinsic fines (see Table 1); in fact the presence of a reduced percentage of fines is usually reported as a factor that could detrimentally affect the aerosolization performance of the corresponding formulation [35,36]. 

The relevance of lactose solid-state was evaluated also by considering its effect on the respirability of a drug with more lipophilic characteristics: budesonide (BUD). Lα·H_2_O and Lα_S_ were used as carriers in the preparation of two binary mixtures, coded as M-BUD and S-BUD, respectively, containing 1% w/w budesonide. Table 5 shows the results of the in vitro deposition tests performed on the prepared mixtures. The mixture prepared with anhydrous α-lactose afforded a FPF of 12% in quite good agreement with the figure reported by Donovan and Smyth [8] for budesonide/anhydrous lactose (though of unspecified anomeric composition) formulations. Similar results were reported by Pitchayajittipong et al. [30] with binary mixtures of budesonide and α-lactose monohydrate or anhydrous β-lactose of comparable intrinsic fines content.

In the present case, the use of an anhydrous carrier (Lα_S_) negatively affected the performance of aerosolization, resulting in a decreased FPF compared to the formulation prepared with Lα·H_2_O. Though in an opposite way to what was observed with salbutamol sulphate, even in this case the nature of lactose solid-state played a key role in determining the interaction between carrier and drug and the final deposition of the latter.

As a first approximation, it appears that the use of a carrier with physico-chemical surface characteristics different from those of the drug in terms of hydrophilicity leads to a reduction of the adhesion between the two components and, thus, to an improved drug detachment and deposition. This has evidently to do with the total surface energy of the carrier, which represent the main thermodynamic driver for the interaction. As a matter of fact, it is well known that different polymorphs or pseudopolymorphs afford different free energy content both at bulk and surface level [37]. The relationship between total energy of the lactose surface and aerosolization performance of micronized drugs is still matter of debate [17,38,39]. However, the understanding of the mechanism beyond the observed phenomena is out of the scope of the present work, which aimed at putting in clear evidence, for the first time, that the well-documented effect of fines on the improvement of the drug deposition could be ascribed not only to the size of the ternary component added to the mixture but also and crucially to the solid-state characteristics of the carrier surface that might stem from common unit operation such as micronization [29].

## 4. Conclusions

This work pointed out that the presence of a fixed amount of fine lactose in lactose/salbutamol sulphate mixtures has a significantly positive effect on the performance of respirability. However, this positive effect decreases and eventually disappears with the aging of the mixtures. This has to be attributed, at least in part, to the solid-state characteristics of lactose fines, whose surface is mainly made up of unstable components, such as hygroscopic anhydrous α-lactose and amorphous lactose, which continuously change over time. These changes affect the interaction between carrier and drug and, consequently, the aerosolization performance.

The key role of lactose solid-state in affecting DPI performance was confirmed by the measurements performed on binary mixtures including different polymorphs of lactose as carriers. Anhydrous forms of α-lactose provided increased deposition of salbutamol sulphate with respect to the conventional α-lactose monohydrate. The opposite tendency was highlighted in the case of budesonide.

On the basis of the obtained results, it was speculated that hydrophilic drugs (such as salbutamol sulphate) would benefit from the use, as carrier, of an anhydrous form of α-lactose, while more lipophilic drugs (such as budesonide) would rather require the use of α-lactose monohydrate.

Although the concomitant effect of lactose surface smoothing and or roughness, anomeric composition, drug surface topography, as well as device de-aggregation mechanism cannot be disregarded, the results reported here strongly underscore the existence of a correlation between the solid-state of α-lactose (employed both as carrier or as fine particles) and the aerosolization performance of a micronized API in a DPI formulation. 

## Figures and Tables

**Table 1:** Particle size distribution parameters of lactose carriers: α-lactose monohydrate, Lα·H_2_O; hygroscopic anhydrous α-lactose, Lα_H_; stable anhydrous α-lactose, Lα_S_. Mean values ± standard deviation (*n* = 3).

Sample	d_V10_ (µm)	d_V50_ (µm)	d_V90_ (µm)
Lα·H_2_O	10.2 ± 0.8	53.1 ± 2.0	103.3 ± 2.6
Lα_H_	18.8 ± 1.1	54.7 ± 0.7	104.9 ± 0.6
Lα_S_	20.7 ± 2.2	56.3 ± 0.4	105.7 ± 1.4

**Table 2:** Composition (% *w*/*w*) of the studied ternary mixtures of α-lactose monohydrate (Lα·H_2_O), salbutamol sulphate (SS), and variable percentages (from 10% to 50%) of micronized lactose in comparison with a binary mixture containing no micronized lactose (M-SS).

Mixture	Lα·H_2_O (%)	Micronized Lactose (%)	SS (%)
M-SS	99	-	1
M-10	89	10	1
M-30	69	30	1
M-50	49	50	1

**Table 3:** In Vitro deposition tests—emitted dose (ED), fine particle dose (FPD), and fine particle fraction (FPF) of salbutamol sulphate from ternary mixtures of α-lactose monohydrate (Lα·H_2_O), salbutamol sulphate (SS), and variable percentages (10%, 30%, and 50% w/w) of micronized lactose in comparison with a binary mixture containing no micronized lactose (M-SS). Mean values ± standard deviation (*n* = 5).

Mixture	ED (µg)	FPD (µg)	FPF (%)
M-SS	177.1 ± 3.4	26.8 ± 1.9	15.1 ± 0.8
M-10	178.7 ± 4.6	84.3 ± 2.6	47.2 ± 0.7
M-30	180.9 ± 9.7	103.4 ± 7.3	57.2 ± 2.5
M-50	102.8 ± 5.5	34.9 ± 4.6	33.9 ± 2.8

**Table 4:** In Vitro deposition tests—ED, FPD, and FPF of salbutamol sulphate (SS) from binary mixtures containing different polymorphs of lactose as carriers: hygroscopic anhydrous α-lactose, H; stable anhydrous α-lactose, S.

Mixture	ED (µg)	FPD (µg)	FPF (%)
H-SS	182.7 ± 11.6	76.2 ± 3.6	41.7 ± 1.9
S-SS	173.0 ± 2.7	54.4 ± 2.2	31.5 ± 0.9

**Table 5:** In Vitro deposition tests—ED, FPD, and FPF of budesonide (BUD) from binary mixtures containing Lα·H_2_O (M) and Lα_S_ (S) as carriers. Mean values ± standard deviation (*n* = 5).

Mixture	ED (µg)	FPD (µg)	FPF (%)
M-BUD	154.1 ± 4.3	27.9 ± 0.1	18.1 ± 0.5
S-BUD	149.6 ± 4.0	17.9 ± 1.3	12.0 ± 0.5
